# Single-crystal neutron and X-ray diffraction study of garnet-type solid-state electrolyte Li_6_La_3_ZrTaO_12_: an *in situ* temperature-dependence investigation (2.5 ≤ *T* ≤ 873 K)

**DOI:** 10.1107/S2052520620016145

**Published:** 2021-01-26

**Authors:** Günther J. Redhammer, Martin Meven, Steffen Ganschow, Gerold Tippelt, Daniel Rettenwander

**Affiliations:** a Department of Chemistry and Physics of Materials, Division of Materials Science and Mineralogy, University of Salzburg, Jakob-Haringerstr. 2A, Salzburg, 5020, Austria; b Institute of Crystallography, RWTH Aachen University, Jaegerstrasse 17/19, Aachen, 52056, Germany; c Jülich Centre for Neutron Science (JCNS), Forschungszentrum Jülich GmbH at Heinz Maier-Leibnitz Zentrum (MLZ), Lichtenbergstrasse 1, Garching, 85748, Germany; d Leibniz-Institut für Kristallzüchtung (IKZ), Max-Born-Strasse 2, Berlin, 12489, Germany; e Institute for Chemistry and Technology of Materials, Graz University of Technology, Stremayrgasse 9, Graz, 8010, Austria

**Keywords:** LLZO-type solid-state electrolyte, single-crystal neutron diffraction, *in situ* temperature dependence, stability on ageing

## Abstract

Large single crystals of garnet-type Li_6_La_3_ZrTaO_12_ (LLZTO) were grown by the Czochralski method and analysed using neutron diffraction between 2.5 and 873 K in order to fully characterize the Li atom distribution, and possible Li ion mobility in this class of potential candidates for solid-state electrolyte battery material. LLZTO retains its cubic symmetry (space group *Ia* 3 *d*) over the complete temperature range. When compared to other sites, the octahedral sites behave as the most rigid unit and show the smallest increase in atomic displacement parameters and bond length. The La and Li sites show similar thermal expansion in their bond lengths with temperature, and the anisotropic and equivalent atomic displacement parameters exhibit a distinctly larger increase at temperatures above 400 K. Detailed inspection of nuclear densities at the Li1 site reveal a small but significant displacement from the 24*d* position to the typical 96*h* position, which cannot, however, be resolved from the single-crystal X-ray diffraction data. The site occupation of Li^I^ ions on Li1 and Li2 sites remains constant, so there is no change in site occupation with temperature.

## Abstract 2

Single-crystal neutron and X-ray diffraction methods were used to evaluate the exact crystal structure, Li site occupation and diffusion path of nominal Li_6_La_3_ZrTaO_12_ at six temperatures between 2.5 K and 873 K.

## 1. Introduction   

Modern and high-performance energy storage devices based on the Li ion battery technology require the choice of a safe electrolyte to overcome known problems with the electrochemical and thermodynamic stability of metallic Li and/or the formation of dendrites when using liquid electrolytes. Such ion-conducting electrolytes should have exceptionally high ionic conductivities at room temperature coupled with negligible electronic conductivities, high stability and resistance to chemical reaction with both the anode and cathode, especially elemental Li, a high electrochemical decomposition voltage and, last but not least, should also be environmentally friendly and of low cost (Thangadurai *et al.*, 2014 ▸; Ramakumar *et al.*, 2017 ▸; Knauth, 2009 ▸; Wang *et al.*, 2020 ▸). Solid (ceramic) electrolytes are proposed to overcome these limitations and facilitate a large step towards all-oxide, solid-state Li batteries.

The garnet family, well known for decades in mineralogy and crystallography (Novak & Gibbs, 1971 ▸), has been identified to be one of a few promising candidates for such demanding applications (Murugan *et al.*, 2007 ▸). Total conductivities of 0.4 m S cm^−1^ were observed at room temperature, *e.g.* in the compound Li_7_La_3_Zr_2_O_12_ (LLZO), however, only in the cubic modification. The latter can be stabilized by doping with aliovalent cations such as Al^III^ (Buschmann *et al.*, 2011 ▸; Geiger *et al.*, 2011 ▸) instead of Li^I^ at the tetrahedral site, thereby introducing a degree of Li disorder. The pure end-member LLZO is tetragonal and has distinctly lower ionic conductivities (Awaka *et al.*, 2009 ▸). A large amount of work has been carried out on the different possible cations and substitutions that can be used to stabilize the cubic modification by replacing Li^I,^ with, for example, Al^III^, Ga^III^, Fe^III^ (Rettenwander *et al.*, 2014 ▸; Rettenwander, Wagner *et al.*, 2016 ▸; Wagner, Redhammer, Rettenwander, Senyshyn *et al.*, 2016 ▸; Wagner, Redhammer, Rettenwander, Tippelt *et al.*, 2016 ▸; Rettenwander, Redhammer *et al.*, 2016 ▸; Wagner *et al.*, 2017 ▸) and references therein.

Replacement of Zr^IV^ by Ta^V^, among others, also stabilizes the garnet in its body-centred cubic modification (Hamao *et al.*, 2017 ▸) and, thereby, also reduces the Li content. While some studies found a stabilization of the cubic phase at low Ta^V^ contents of *x* = 0.125 (Logéat *et al.*, 2012 ▸) or *x* = 0.2 (Gong *et al.*, 2019 ▸; Yi *et al.*, 2019 ▸) along the Li_7–*x*_La_3_Zr_2–*x*_Ta_*x*_O_12_ series, a phase-pure cubic phase is reported to occur above *x* = 0.6 in Al-free environments (Wang & Lai, 2015 ▸; Hamao *et al.*, 2017 ▸). It has been shown that at Li contents of around 6.5 formula units, the cubic modification undergoes a symmetry reduction to its tetragonal form (Thompson *et al.*, 2015 ▸, 2014 ▸). LLZOs, substituted with Ta^V^, are of special interest as they have among the highest ionic conductivities of any Li garnet-type materials and, as such, have a wide range of potential applications. Consequently, the system Li_7–*x*_La_3_Zr_2–*x*_Ta_*x*_O_12_ has been the subject of several studies (Hamao *et al.*, 2016 ▸; Wang *et al.*, 2014 ▸, 2017 ▸; Wang & Lai, 2012 ▸). Recently, ionic conductivities as high as 1.39 × 10^−3^ S cm^−1^ at room temperature for *x* = 0 have been reported for five large single crystals of Li_6.5_La_3_Zr_1.5_Ta_0.5_O_12_ and Li_7–*x*_La_3_Zr_2–*x*_Nb_*x*_O_12_ (Kataoka & Akimoto, 2018 ▸; Kataoka *et al.*, 2018 ▸). The high ionic conductivity is explained by a small positional disorder of the normally tetragonally coordinated Li at the 24*d* position, which is split into four partially occupied 96*h* positions (Kataoka *et al.*, 2018 ▸).

This study further investigates these initial findings with an in-depth look at the crystal structure of high-quality Czochralski-grown single crystals of Li_6_La_3_ZrTaO_12_ (Stanje *et al.*, 2017 ▸) over a wide temperature range, with special emphasis on the Li distribution, using combined single-crystal neutron and X-ray diffraction methods. A companion paper deals with the stability of LLZTO in the presence of moisture and different wet environments such as distilled water and acetic acid (Redhammer *et al.*, 2021 ▸).

## 2. Experimental   

### 2.1. Synthesis   

**Figure 1:**
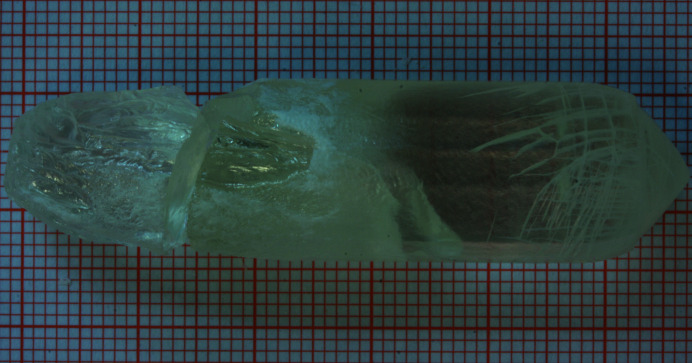
As-grown Li_6_La_3_ZrTaO_12_ crystal. Strong evaporation of Li-containing species from the melt, and possibly also from the crystal, cause serious corrosion of the growing crystal, especially in its upper, first-grown part (left side in the photograph). Most affected is the seed crystal that usually breaks during cooling down to room temperature. Growth direction close to [100].

For the neutron diffraction experiment, a single crystal was grown using the Czochralski pulling technique. The starting material for growth experiments comprised a mixture of dry 50 mol% Li_2_CO_3_, 25 mol% La_2_O_3_, 16.7 mol% ZrO_2_ and 8.3 mol% Ta_2_O_5_ (corresponding to a sum formula Li_6_La_3_ZrTaO_12_) with an additional excess of 20 mol% Li_2_O. The mixed powders were pressed isostatically at 500 bar (1 bar = 10^5^ Pa), fired for 6 h at 850°C, ground, pressed at 2000 bar and fired again for 6 h at 1230°C, before being melted in an inductively heated 40 ml iridium crucible with alumina ceramic insulation. After a period of melt homogenization, a randomly oriented seed crystal, obtained in a previous experiment, was added to initiate crystallization. A 50 mm × 15 mm crystal was pulled at a rate of 0.4 mm h^−1^ using automatic diameter control of the growth apparatus. After the growth was completed, the crystal was withdrawn from the melt and cooled to room temperature over 15 h. The whole growth process was carried out in a pure N_2_ atmosphere. The as-grown crystal (Fig. 1 ▸) was cut perpendicular to the growth direction and slabs and cuboids ∼ 2.0–2.5 mm in length were prepared for various analytical techniques from the clear and flawless parts of the ingot. Chips from the central clear part of the crystal were gently crushed to reduce the crystallite size to around 0.1–0.3 mm, which were then used for single-crystal X-ray diffraction measurements between 90 K and 300 K.

### 2.2. Single-crystal X-ray diffraction (SCXRD)   

Single-crystal X-ray diffraction data were collected on a Bruker SMART APEX CCD diffractometer. The single crystals were selected on the basis of their optical properties (sharp extinction, regular shape and homogeneity in colour) and glued on a glass capillary (0.1 mm in diameter). Intensity data were collected using graphite-monochromated Mo *K*α X-ray radiation (50 kV, 20 mA) with an ω-scan mode set-up at four different φ positions (0°, 90°, 180° and 270°). A total of 665 frames with Δω = 0.3° were acquired for each run. Three-dimensional data were integrated and corrected for Lorentz, polarization and background effects using *APEX2* software (Bruker, 2012 ▸). Structure solution using direct methods and subsequent weighted full-matrix least-squares refinements on *F* ^2^ were carried out with *SHELX-2012* (Sheldrick, 2015 ▸) incorporated in the program suite *WinGX 2014.1* (Farrugia, 2012 ▸).

Analysis of X-ray diffraction intensity data sets for samples sometimes showed the presence of weak superstructure reflections that obey the 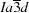 symmetry, but indicate a primitive unit cell. A similar phenomenon was described by Kataoka *et al.* (2018 ▸) for their LLZTa_0.5_O single crystals, which were of a similar size (∼0.15 mm) to those used in this study. To judge whether the superstructure reflections could be artefacts from the data collection methodology, the crystals were reanalysed with reduced operation voltages of the X-ray tube (30 kV instead of 50 kV) and longer integration times (to have comparable intensities on the main peaks); in these experiments λ/2 radiation was not stimulated. Indeed, the peaks corresponding to the body-centred cell are absent in the reduced generator power experiments. Thus, it is concluded that, at least in our experiments, the additional observed Bragg peaks in some of the investigated samples can be assigned to λ/2 contributions, a phenomenon which is especially found in area detector data and for *I* body-centred cells (Kirschbaum *et al.*, 1997 ▸; Gianopoulos *et al.*, 2017 ▸; Secco *et al.*, 2008 ▸).

### 2.3. Single-crystal neutron diffraction   

Single-crystal neutron diffraction intensity data were collected on the HEiDi diffractometer at temperatures between 2.5 and 830 K with λ = 0.793 Å [Ge(420) monochromator]. HEiDi is a four-circle, single-crystal diffractometer with an acentric Eulerian cradle and a high flux of hot neutrons from beam tube SR9B from the FRM II reactor at the Heinz Maier-Leibnitz Zentrum (Meven & Sazonov, 2015 ▸). Data acquisition was carried out up to sinθ/λ = 0.808 Å^−1^ with about 675 Bragg reflections to ensure full structure characterization. The *WinGX* (Farrugia, 2012 ▸) package of programs and *SHELXL2014* (Sheldrick, 2015 ▸), in combination with the *FullProf* suite for combined simultaneous refinement of single-crystal X-ray (SCXRD) and single-crystal neutron diffraction (SCND) analysis and bond-valence sum map display, were used for full structural analysis.

Details on data collection, crystal data and refinements at selected temperatures for the neutron data and sample treatment procedures of the single crystals are given in Table 1 ▸. Selected structural parameters are compiled in Table 2 ▸, whereas the atomic coordinates and equivalent atomic displacement parameters can be found in supplementary Table S1. The anisotropic atomic displacement parameters are accessible from the CIF. Analysis of possible ion-diffusion pathways and estimation of migration energy barriers was carried out using *BondStr* (Katcho *et al.*, 2019 ▸) and *SoftBV* (Chen *et al.*, 2019 ▸). Maximum entropy method (MEM) reconstructions of the nuclear densities were performed using *Dysnomia*, within the *FullProf* suite. Structural drawings were constructed using *VESTA* (Momma & Izumi, 2011 ▸).

## 3. Results and discussion   

### 3.1. Structure model building of Li_6_La_3_ZrTaO_12_   

**Figure 2:**
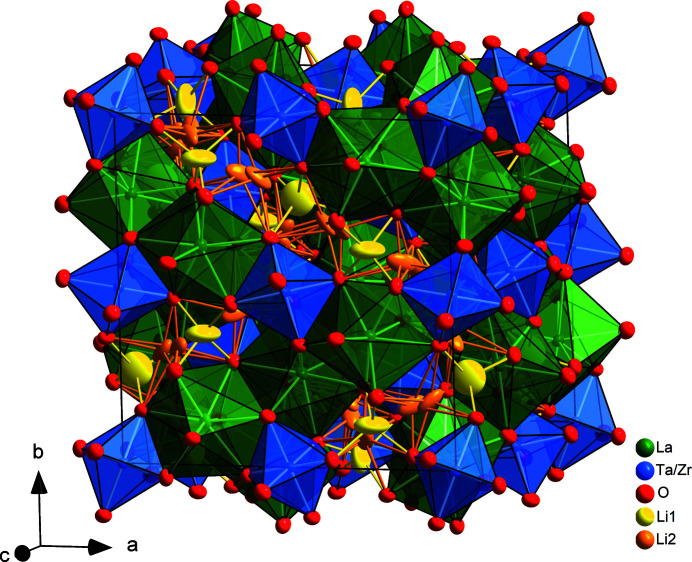
Polyhedral representation of the crystal structure of nominal La_6_La_3_ZrTaO_12_ at 300 K determined from the neutron diffraction data. Anisotropic atomic displacement parameters are shown at the 95% probability level. The LaO_8_ sites are green and Zr/TaO_6_ octahedra are blue. The Li1 (yellow) and Li2 (orange) sites are shown as anisotropic atomic displacement ellipsoids only to highlight the three-dimensional network for Li ion conduction.

Neutron diffraction data on a large piece of the Czochralski grown LLZTO (CZ-LLZTO) crystal were collected at 2.5 K, 200 K, 300 K, 400 K, 573 K and 873 K. At all temperatures, the analysis of systematic extinctions yields the space group 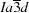, and no systematic absence violations were observed. Unfortunately, the crystal started to develop some cracks which caused small disorientations of the fragments at 573 K and more severely at 873 K, so the higher temperature data sets are considered to be of an inferior quality. The 300 K data were used for full structure determination using direct methods, and the Li position was determined from analysis of negative residual nuclear densities in multiple Fourier maps. LLZTO adopts the typical garnet structure. La^III^ ions reside on 24*c* sites and are eightfold coordinated with oxygen atoms, with two different La—O bond distances of 2.4945 (5) and 2.5940 (5) Å. A small deficit of La^III^ on this site is in accordance with a more general finding of a small deficit in site occupation in previous published studies of our working group (Wagner, Redhammer, Rettenwander, Senyshyn *et al.*, 2016 ▸; Wagner, Redhammer, Rettenwander, Tippelt *et al.*, 2016 ▸). Zr^IV^/Ta^V^ ions occupy 16*a* sites, which have a regular octahedral coordination with Zr/Ta—O bond length of 2.0446 (5) Å. One octahedron shares its edges with six neighbouring dodecahedral sites, thereby forming an open, integrated framework, in which the Li atoms are positioned. A display of the crystal structure is given in Fig. 2 ▸.

The Fourier maps (note, only the framework atoms La, Zr/Ta and O were used in the refinement) revealed two strong negative nuclear densities corresponding to the two Li positions, which supports the model generally assumed for 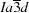 LLZO material as reported by Stanje *et al.* (2017 ▸). Li1 occupies the regular tetrahedrally coordinated 24*d* site at (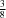, 0, ¼) of the garnet structure (normally occupied by Si^IV^ in silicate garnets), and Li2 resides at an interstitial site at usual 96*h* position with coordinates of 0.15, 0.17, 0.44. Both Li atomic positions show distinct anisotropic atomic displacement parameters with Li1 showing slightly larger vibrations; the principal mean-square atomic displacement *U*_max_/*U*_min_ include values of 4.46 and 4.06 for Li1 and Li2, respectively, indicative of distinct (thermal atomic) motion of the Li atoms in the dodecahedral–octahedral framework. It is worth noting that no significant residual nuclear densities can be detected in the different Fourier syntheses of the final model. Therefore, it is clear that Li ions occupy only these two positions in LLZTO, which is in contrast to some previous work (Wang *et al.*, 2020 ▸; Thangadurai *et al.*, 2014 ▸) and suggests that 48*g* also hosts Li ions.

**Figure 3:**
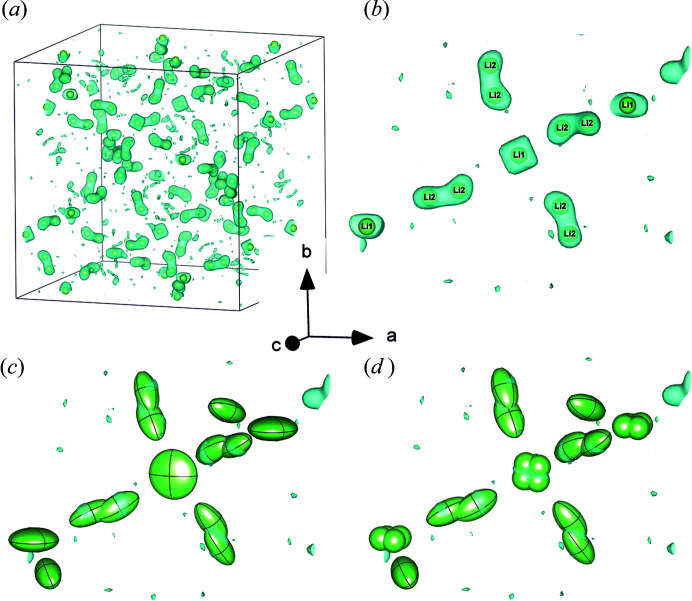
Negative residual nuclear density (light bluish green) of LLZTO at 300 K, obtained from refining the framework La, Zr/Ta and O atom positions, for (*a*) the full unit cell and (*b*) a small section of the cell. The spheres depict the refined Li atomic positions using; (*b*) the standard model, (*c*) the model with anisotropic refinement of Li1 at 24*d* with atomic displacement parameters, drawn at the 95% level, and (*d*) the model with Li1 shifted to a 96*h* position and isotropic refinement.

In detail, the negative residual nuclear density at the Li1 site is less spherical and is more square shaped than that at the Li2 site. This is interpreted to be a manifestation of a small displacement of Li atoms away from 24*d* towards the usual 96*h* position (Fig. 3 ▸). The very same observation was also recently reported by Kataoka & Akimoto (2018 ▸) for the Li1 position in Li_6.5_La_3_Zr_1.5_Ta_0.5_O_12_ based on neutron diffraction on a large single crystal.

This shift from 24*d* to 96*h* is small but refinable, and amounts to 0.248 (18) Å, yielding atomic positions for Li1 of 0.3739 (14), −0.012 (3), 0.235 (2) at 300 K. However, in shifting this position, only an isotropic refinement of the atomic displacement parameters for Li1 is possible at any temperature tested (Fig. 3 ▸ *d*). In this ‘shift’ model, the Li1 atoms are displaced from each other by 0.360 (11) and 0.509 (15) Å at 300 K, allowing shorter Li1⋯Li2 jump distances of 1.398 (14) Å and 1.63 (4) Å, respectively. The Li2⋯Li2 distance remains at 0.72 (13). The corresponding values in the 24*d* model are 1.628 (6) and 0.722 (11) Å for Li1⋯Li2 and Li2⋯Li2, respectively. So, the shift model would allow for shorter possible jump distances. The (isotropic) 96*h* position model and the 24*d* position (anisotropic) model for Li1 yield equally good refinements as both fit the observed negative nuclear densities similarly well [Figs. 3 ▸(*c*) and 3 ▸(*d*)]. In single-crystal X-ray diffraction data refinement, however, the refinements of the shifted position are unstable, most probably due to the low scattering power of Li. Regardless, for the purpose of comparison, the widely applied model with Li1 at 24*d* is preferred in the further discussion below.

**Figure 4:**
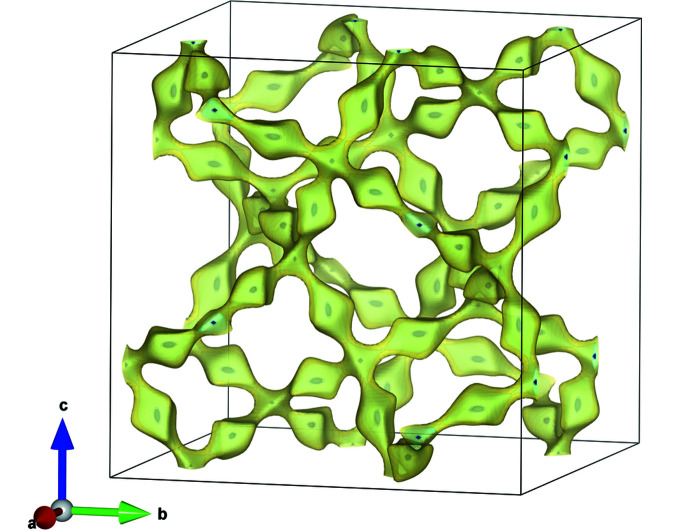
Bond valence energy landscape map at levels of 0.1 eV (dark blue) and 0.5 eV (yellow) above the minimum. A three-dimensional network for Li ion mobility is evident. Observed Li positions coincide with calculated energy minima.

Bond valence energy landscape (BVEL) map calculations (Chen *et al.*, 2019 ▸; Chen & Adams, 2017 ▸; Adams & Rao, 2014 ▸) were applied to investigate possible ion diffusion pathways. In the calculated three-dimensional network for Li^I^ ion migration in LLZTO, the Li^I^ positions fit very well with the energy minima in the BVEL map (Fig. 4 ▸). Also, an activation energy for Li ion jumps is calculated to be 0.35 eV, which is in good agreement with published values (Stanje *et al.*, 2017 ▸).

The Zr/Ta value of the studied CZ-LLZTO crystal departs from the ideal 1:1 ratio as has already been noted by Stanje *et al.* (2017 ▸). As the nuclear bound scattering lengths of Zr and Ta are similar in neutron diffraction (7.16 and 6.91 fm respectively), the final site distribution of the two elements on the octahedrally coordinated 16*a* site was determined by simultaneous refinement of both the 300 K SCND and SCXRD data (with slightly more weight given to the neutron diffraction data). The result was a cation distribution of Zr_0.90_Ta_1.10_, which was then fixed in the refinements of neutron data for all other temperatures. Due to its sensitivity to Li ions, the neutron diffraction data provided very reliable Li ion site occupation. Significant vacancies are present at both Li sites; the Li1 site is only two-thirds filled and the interstitial 96*h* position shows an occupation of only one-third. The refinements on the SCND data alone yield similar values to those from the simultaneous refinement for Li occupation at the Li1 and Li2 sites, *i.e.* 0.676 (19) and 0.351 (8) compared to 0.659 (19) and 0.341 (7), respectively. On applying the latter values in addition to the slight deficit on the La site, which is also revealed in the simultaneous refinement, a charge balanced formula of Li_6.07_La_2.95_Zr_0.90_Ta_1.10_O_12_ is obtained. Using the ‘standard’ model with unsplit 24*d* site allows all atoms to be refined with their anisotropic atomic displacement parameters up to 573 K. The resultant reliability factors are low and attest to the high quality of the data and refinements.

### 3.2. The structure of Li_6_La_3_ZrTaO_12_ between 2.5 K and 873 K   

**Figure 5:**
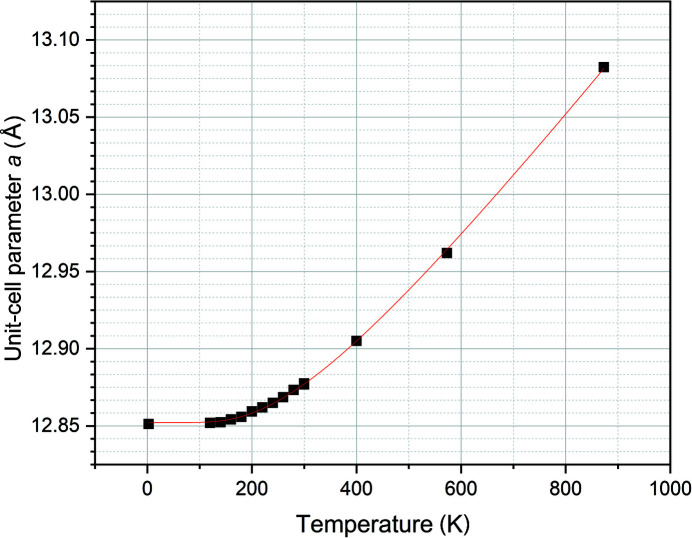
Variation of unit-cell parameter *a* with temperature, as determined from single-crystal neutron and X-ray diffraction data. The line is fitted to the data using the Salje equation (Salje *et al.*, 1991 ▸) as described in the text.

The unit-cell parameters, determined for the temperatures between 120 K and 300 K in the neutron diffraction experiments using single-crystal X-ray diffraction, overlap. Saturation of the thermal expansion is observed at low temperature. This can be addressed when fitting the data (Fig. 5 ▸) by using the equation suggested by Salje *et al.* (1991 ▸) that is implemented in *EosFit7* (Gonzalez-Platas *et al.*, 2016 ▸);
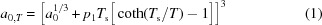
Here, 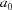 corresponds to the unit-cell parameter *a* at the reference temperature *T*_ref_ = 0 K, *T*_s_ is saturation temperature above which the thermal expansion becomes constant. The value of *p*_1_ is described to be approximately three times the high-temperature value of the thermal expansion coefficient α. Using this equation, *a*_0_ = 12.8522 (5) Å, *p*_1_ = 14.50 (16) with α ∼ 4.8 × 10^−5^ and a saturation temperature *T*_s_ of 409 (7) K.

**Figure 6:**
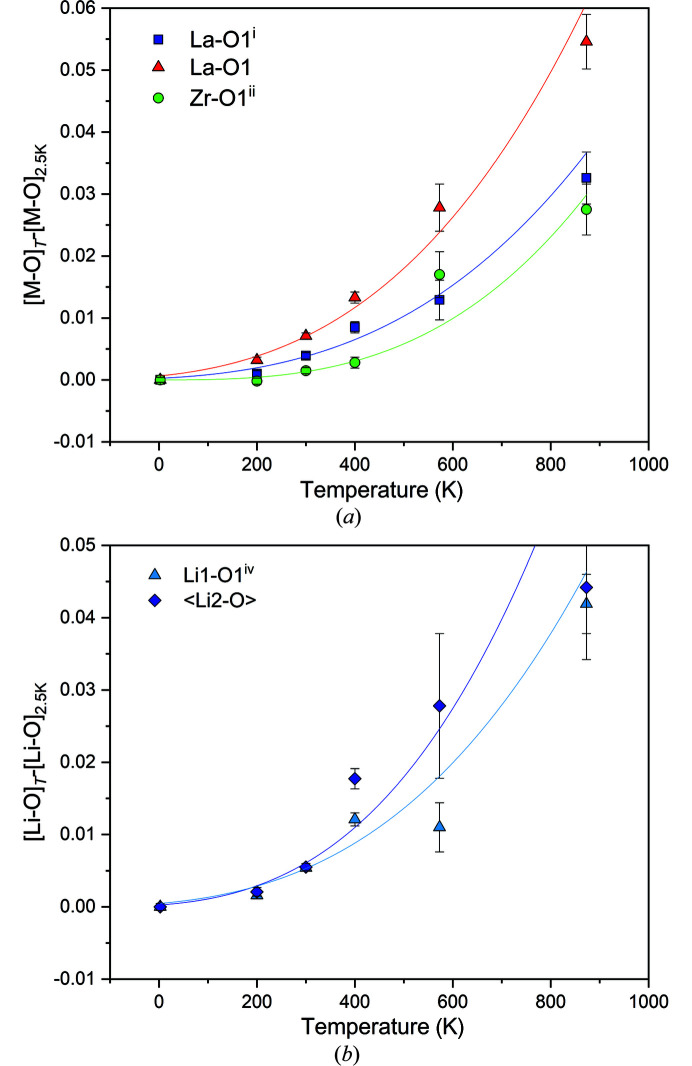
Changes in bond lengths in nominal Li_6_La_3_ZrTaO_12_ relative to the value at 2.5 K, as a function of temperature. Data were fitted using the Salje equation (Salje *et al.*, 1991 ▸), regression lines serve only as a visual guide. Error bars are not visible where the error is smaller than the symbol.

With increasing temperature, the dodecahedral site shows a larger thermal expansion than the octahedral site. La—O1 and La—O1 bond lengths increase between 2.5 K and 800 K by 0.032 and 0.056 Å, respectively, and the Zr/Ta—O1 bonds expand by 0.028 Å (Table 2 ▸ and Fig. 6 ▸). The equivalent thermal displacement parameter, *U*_eq_, also is lowest for the Zr/Ta at the octahedral site, and increases gradually with increasing temperature (Fig. 6 ▸). A very similar behaviour is shown by the oxygen atom; *U*_eq_ values are intermediate between Zr/Ta, but O atoms show a more marked non-linear increase. The low temperature *U*_eq_ values are close to those for Zr/Ta, but are more similar to those for the oxygen atom at higher *T*. The La atoms, in particular, show some distinct anisotropic thermal motion, with a *U*_max_/*U*_min_ ratio at 2.5 K of 3.12, 1.77 and 1.58 for La, Zr/Ta and O atoms, respectively. Interestingly, the anisotropy of these three atoms decreases as temperature increases, with principal square atomic displacements *U*_max_/*U*_min_ at 400 K of 1.71, 1.18 and 1.67 for La, Zr/Ta and O atoms, respectively. The single-crystal X-ray diffraction data show the very same trends. However, there is more scatter in the displacement parameters and bond lengths.

In the unsplit model, the 24*d* Li1 site corresponds to the regular tetrahedral site of the garnet structure. In silicate garnets, the tetrahedron typically remains very rigid during temperature change, which is in contrast to what is observed here. The Li1—O bond lengths increase slowly at low temperatures from 1.9169 (5) Å at 2.5 K to 1.9291 (9) at 400 K, and then more rapidly to 1.959 (4) Å at 800 K, with associated increases in the polyhedral volume of 2.7% in the 2.5 K to 400 K range and 14% up to 800 K. These changes must be due to shifts in the oxygen positions, as Li resides on a fixed 24*d* position. Unfortunately, the Li—O bond length, in particular, suffered larger estimated standard uncertainties and uncertainties due to the breaking of the crystal during data collection. Nevertheless, the data from Li1—O^i^ (i = *z*, *x*, *y*) fit a thermal expansion trend well as would, perhaps, be expected from the fit of the data with the thermal expansion model of Salje *et al.* (1991 ▸). The separation of Li1–Li2 also changes with *T*: the shortest distance is 1.628 (6) Å at 2.5 K. It then increases slowly by 0.006 Å with temperature towards 300 K, above which it then extends by ≃0.06 Å nearing 800 K. In contrast, the longer Li1–Li2 interatomic distance increases by only ≃ 0.015 Å over the whole *T* range. The separation of the Li2–Li2 sites remains constant within the estimated standard deviation. It should be recalled that the simultaneous occupation of both positions is not possible. However, jumps between the two positions are to be expected, and probably have low activation energies. The Li2 site can also be regarded as fourfold coordinated, with bond lengths ranging between 1.858 (6) Å and 2.280 (5) Å at 2.5 K, with an average 〈Li2—O〉 bond length of 2.089 (6) Å. Two more distant oxygen atoms are 2.654 (5) and 2.675 (5) Å away from Li2. As temperature increases, the Li2—O bond lengths generally also increase, although the trends are not smooth and there is some scatter. On average, 〈Li2—O〉 increases by ≃ 2.1% to 2.133 (9) Å at 800 K, but it is important to note that some of the individual Li2—O bond lengths change more than others (*e.g.* the shortest Li2—O distance increases by 0.065 Å). An even larger stretching of the 〈Li2—O〉 bonds might be expected when assuming a constant thermal expansion at high temperature (Fig. 6 ▸ *b*).

**Figure 7:**
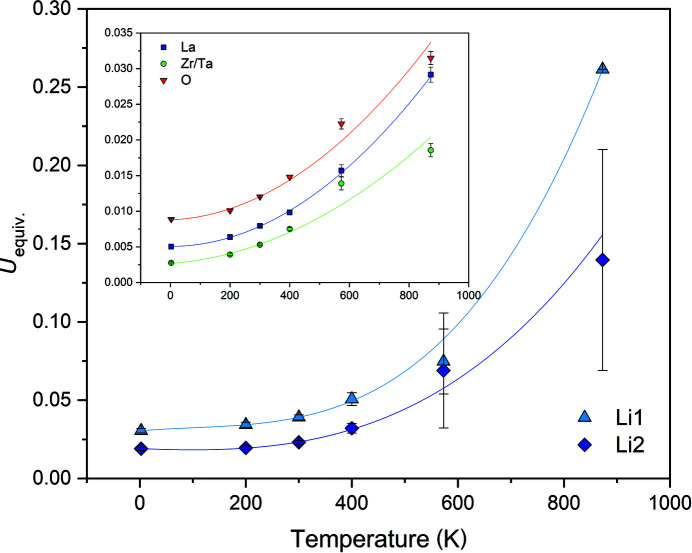
Variation of equivalent isotropic atomic displacement parameters for nominal Li_6_La_3_ZrTaO_12_ with temperature, as determined from neutron diffraction data. Third order polynomials were fitted to the data and serve as visual guides. Error bars are not visible where the error is smaller than the symbol.

Over the entire temperature range, the Li1 site at 24*d* exhibits the largest equivalent atomic displacement parameter *U*_eq_, which distinctly increases with increasing temperature. Furthermore, there is a much steeper increase above 400 K when compared to the change at the Li2 site and especially so when compared to the changes associated with the La, Zr/Ta and O atoms (Fig. 7 ▸). This may indicate that there is, indeed, some shift at the Li1 site from the special to the general 96*h* position, and that Li is highly mobile at high temperatures. Also, the increase of *U*_eq_ at the Li2 site is steeper than for the framework atoms. Generally, both Li sites appear to be highly anisotropic even at the lowest temperature of 2.5 K with *U*_max_/*U*_min_ ratios of 5.5 and 4.6 for Li1 and Li2, respectively. These ratios do not change much with increasing temperature. The largest elongation of the thermal ellipsoids follows the direction of the most probable Li ion mobility pathway within the channels of minimum energy (*cf*. Fig. 4 ▸).

**Figure 8:**
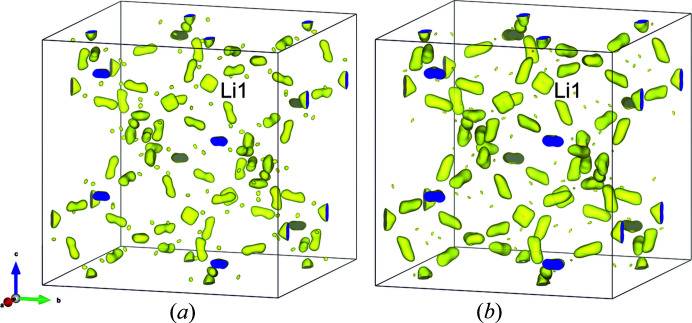
Comparison of the negative nuclear density maps as obtained from MEM reconstruction using experimental structure factors in Li at 2.5 K (left) and 400 K (right). Note, the elongate and square-like shapes of the nuclear densities at Li1 sites in the MEM reconstructions.

An increase in thermal motion is also visible in the reconstruction of the nuclear density maps from the experimental structure factors, using the maximum entropy method as depicted in Fig. 8 ▸. It is evident that at 2.5 K, Li ions at Li1 and Li2 are very focused, but with increasing *T*, the nuclear densities at Li2 become more elongated or adapt a more pronounced square-like shape. Unfortunately, the MEM data collected at higher *T* are of insufficient quality to be useful. Nevertheless, it is worth noting that the site occupation factors at Li1 and Li2 sites remain remarkably constant, so there is no evident change in the distribution of Li^I^ atoms over the two possible sites as temperature changes.

Finally, some comment on the split model should be given: there is an increased shift from the 24*d* position with increasing temperature. The shift is 0.246 (13) Å at 2.5 K and remains similar at 300 K, but increases to 0.307 (16) Å at 400 K and 0.42 (3) at 523 K. The Li1⋯Li1 distances in the split model increase accordingly with 0.43 (2) and 0.61 (2) Å recorded at 400 K. The Li1⋯Li2 distances also increase from 1.473 (13) and 1.492 (13) at 2.5 K to 1.57 (2) and 1.91 (6) Å at 573 K.

## 4. Conclusion   

Very accurate structural data for Li_6_La_3_ZrTaO_12_ garnet-type material have been obtained from the neutron diffraction analyses: the Li atom location, including site occupation factors and atomic displacement parameters. Detailed inspection of nuclear density data reveals some displacement of the tetrahedrally coordinated Li1 site from the 24*d* position to the more typical 96*h* position. However, this displacement is small and cannot be resolved in the X-ray diffraction data on the very same material, however, it may explain fast ion transport due to possible shorter jump distances. No indications were found to support Li occupation of a site other than 24*d* and 96*h*. Finally, and most importantly, from the high-resolution single crystal data it was found that, although the anisotropic atomic displacement increases distinctly with temperature, the site occupation of Li1 and Li2 sites remains almost constant. Using powder diffraction data, this is not fully resolvable due to limited resolution and the high correlation of occupation numbers and atomic displacement parameters. In other words, there is no evident migration or change in average site occupation with temperature change. Since no change in the Li ion distribution and no additional sites get occupied by increasing temperature we conclude that the predominating diffusion pathways in LLZTO remain between the 24*d* and 96*h* sites in the Li_6_La_3_ZrTaO_12_ garnet framework.

## Supplementary Material

Crystal structure: contains datablock(s) global, 2.5K, 200K, 300K, 400K. DOI: 10.1107/S2052520620016145/ra5085sup1.cif

Structure factors: contains datablock(s) 2.5K. DOI: 10.1107/S2052520620016145/ra50852.5Ksup2.hkl

Structure factors: contains datablock(s) 200K. DOI: 10.1107/S2052520620016145/ra5085200Ksup3.hkl

Structure factors: contains datablock(s) 300K. DOI: 10.1107/S2052520620016145/ra5085300Ksup4.hkl

Structure factors: contains datablock(s) 400K. DOI: 10.1107/S2052520620016145/ra5085400Ksup5.hkl

Supporting information file. DOI: 10.1107/S2052520620016145/ra5085sup6.pdf

CCDC references: 2049794, 2049795, 2049796, 2049797

## Figures and Tables

**Table 1:** Experimental details and results of refinement of neutron diffraction data between 2.5 K and 400 K for the Czochralski-grown (CZ) LLZTO single crystal For all structures: *M*_r_ = 932.8, cubic, 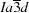, *Z* = 8, crystal dimensions (mm): 2.35 × 2.15 × 1.95. Experiments were carried out with neutron radiation, λ = 0.793 Å, using a Huber Eulerian Cradle diffractometer. Refinement was on 32 parameters with two restraints (occupation of 16*a* site was fixed to full occupation, but Ta/Zr ratio was allowed to refine and in the final refinements, the total Li content was fixed to the value obtained at 2.5 K for all temperatures, but the distribution of Li^+^ over Li1 and Li2 sites was refined freely).

	CZ-LLZTO at 2.5 K	CZ-LLZTO at 200 K	CZ-LLZTO at 300 K	CZ-LLZTO at 400 K
Crystal data				
Chemical formula	Li_6_La_3_O_12_TaZrO_12_	Li_6_La_3_O_12_TaZrO_12_	Li_6_La_3_O_12_TaZrO_12_	Li_6_La_3_O_12_TaZrO_12_
Temperature (K)	2.5	200	300	400
*a* (Å)	12.8511 (2)	12.8592 (2)	12.8775 (2)	12.9051 (2)
*V* (Å^3^)	2122.37 (10)	2126.39 (10)	2135.48 (10)	2149.24 (10)
μ (mm^−1^)	0.04	0.03	0.03	0.03

Data collection				
No. of measured, independent and observed [*I* > 2σ(*I*)] reflections	624, 347, 289	676, 404, 289	674, 401, 291	645, 408, 267
*R*_int_	0.044	0.043	0.028	0.043
(sin θ/λ)_max_ (Å^−1^)	0.811	0.810	0.809	0.811

Refinement				
*R*[*F* ^2^ > 2σ(*F* ^2^)], *wR*(*F* ^2^), *S*	0.023, 0.038, 0.98	0.027, 0.041, 0.97	0.026, 0.033, 1.01	0.046, 0.065, 1.35
No. of reflections	347	404	401	408
Δρ_max_, Δρ_min_ (e Å^−3^)	0.53, −0.40	0.59, −0.64	0.39, −0.49	0.99, −0.97

Computer programs: Bruker *APEX2* (Bruker, 2012 ▸), *SHELXL* (Sheldrick, 2015 ▸), *ORTEP for* Windows (Farrugia, 2012 ▸), *WinGX* (Farrugia, 2012 ▸) publication routines.

**Table 2:** Selected bond lengths and interatomic distances for nominal Li_6_La_3_ZrTaO_12_, as determined from neutron diffraction on CZ-single crystal

*T* = 2.5 K			
La1—O1^i^	2.4906 (5)	Li2—O1^vi^	1.858 (6)
La1—O1	2.5869 (5)	Li2—O1^vii^	2.086 (5)
Zr1—O1^ii^	2.0431 (4)	Li2—O1^viii^	2.280 (5)
Li1⋯Li2^iii^	1.628 (6)	Li2⋯Li1^ix^	2.319 (6)
Li1—O1^iv^	1.9170 (5)	Li2—O1^x^	2.654 (5)
Li1⋯Li2^v^	2.319 (6)	Li2—O1^xi^	2.675 (5)
Li2⋯Li2^vi^	0.722 (11)		

*T* = 200 K			
La1—O1^i^	2.4916 (5)	Li2—O1^vi^	1.856 (6)
La1—O1	2.5901 (5)	Li2—O1^vii^	2.092 (5)
Zr1—O1^ii^	2.0429 (5)	Li2—O1^viii^	2.284 (6)
Li1⋯Li2^iii^	1.631 (6)	Li2⋯Li1^ix^	2.318 (6)
Li1—O1^iv^	1.9185 (5)	Li2—O1^x^	2.657 (6)
Li1⋯Li2^v^	2.318 (6)	Li2—O1^xi^	2.670 (5)
Li2⋯Li2^vi^	0.715 (11)		

*T* = 300 K			
La1—O1^i^	2.4945 (5)	Li2—O1^vi^	1.851 (6)
La1—O1^vi^	2.5940 (5)	Li2—O1^vii^	2.097 (5)
Zr1—O1^ii^	2.0446 (5)	Li2—O1^viii^	2.297 (5)
Li1⋯Li2^iii^	1.634 (6)	Li2⋯Li1^ix^	2.322 (6)
Li1—O1^iv^	1.9224 (4)	Li2—O1^x^	2.671 (5)
Li1⋯Li2^v^	2.322 (6)	Li2—O1^xi^	2.672 (5)
Li2⋯Li2^vi^	0.715 (10)		

*T* = 400 K			
La1—O1^i^	2.4991 (9)	Li2—O1^vi^	1.879 (14)
La1—O1^xii^	2.6002 (9)	Li2—O1^vii^	2.112 (10)
Zr1—O1^ii^	2.0459 (9)	Li2⋯Li1^ix^	2.296 (14)
Li1⋯Li2^iii^	1.668 (15)	Li2—O1^viii^	2.316 (12)
Li1—O1^iv^	1.9291 (9)	Li2—O1^x^	2.653 (13)
Li1⋯Li2^v^	2.296 (14)	Li2—O1^xi^	2.661 (10)
Li2⋯Li2^vi^	0.66 (2)		

Symmetry code(s): (i) *z*, *x*, *y*; (ii) *x* − 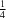, *z* − 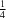, *y* − 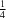; (iii) −*y* + 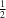, *z* − 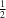, *x*; (iv) −*z* + 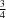, −*y* + 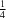, *x* + 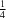; (v) −*z* + 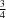, *y* − 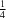, −*x* + 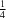; (vi) −*x* + 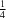, *z* − 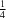, *y* + 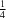; (vii) −*y* + 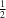, −*z* + 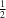, −*x* + 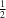; (viii) *y*, −*z* + 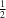, *x* + 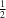; (ix) *y*, *z*, *x*; (x) −*y* + 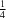, *x* + 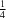, −*z* + 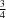; (xi) *y* − 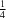, −*x* + 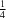, −*z* + 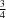; (xii) *x*, −*y*, −*z* + 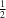.
