# Supplementary material for: Single-crystal neutron and X-ray diffraction study of garnet-type solid-state electrolyte Li6La3ZrTaO12: an in situ temperature-dependence investigation (2.5 ≤ T ≤ 873 K)
Source: Acta Crystallogr B Struct Sci Cryst Eng Mater. 2021 Jan 26;77(Pt 1):123–30. doi: 10.1107/S2052520620016145 (PMC7941284; doi:10.1107/S2052520620016145)
Supplement: Supplementary file 6 [file b-77-00123-sup6.pdf]

STRUCTURAL SCIENCE
CRYSTAL ENGINEERING
MATERIALS

Volume 77 (2021)

Supporting information for article:

Single-crystal neutron and X-ray diffraction study of garnet-type solid-state electrolyte Li6La3ZrTaO12: an in-situ temperature dependence investigation (2.5 K ≤ T ≤ 873 K)

Günther J. Redhammer, Martin Meven, Steffen Ganschow, Gerold Tippelt and Daniel Rettenwander

Table S1 Fractional atomic coordinates, site occupation factor (sof), and equivalent isotropic atomic displacement parameters U_{eq} for $Li_6La_3ZrTaO_{12}$ at selected temperatures.

Atom	site	*x*	*y*	*z*	*sof*	U_{eq}
CZ-LLZO, T = 2.5K, $Ia\bar{3}d$, Z = 8, *a* = 12.8511(2) Å, „24*d*“ standard model						
La1	24*c*	0.125	0	0.25	1.000(5)	0.00504(17)
Zr1	16*a*	0	0	0	0.440(3)	0.00276(13)
Ta1	16*a*	0	0	0	0.560(3)	0.00276(13)
O1	96*h*	0.10301(3)	0.19774(4)	0.28062(4)	1	0.00889(12)
Li1	24*d*	0.375	0	0.25	0.642(5)	0.0305(15)
Li2	96*h*	0.1516(4)	0.1748(4)	0.4376(4)	0.3455(14)	0.0190(11)
CZ-LLZO, *T* = 2.5K, $Ia\bar{3}d$, Z = 8, *a* = 12.8511(2) Å, shift 96*h* model						
La1	24*c*	0.125	0	0.25	1.003(6)	0.0051(2)
Zr1	16*a*	0	0	0	0.451(3)	0.00273(15)
Ta1	16*a*	0	0	0	0.549(3)	0.00273(15)
O1	96*h*	0.10300(4)	0.19772(4)	0.28060(4)	1	0.00886(14)
Li1	96*h*	0.365(2)	-0.006(3)	0.236(3)	0.152(10)	0.012(7)
Li2	96*h*	0.1518(5)	0.1747(5)	0.4375(5)	0.360(12)	0.0196(18)
CZ-LLZO, *T* = 200 K, $Ia\bar{3}d$, Z = 8, *a* = 12.8592(2) Å,						
La1	24*c*	0.125	0	0.25	0.987(5)	0.00638(17)
Zr1	16*a*	0	0	0	0.449(3))	0.00391(14)
Ta1	16*a*	0	0	0	0.551(3)	0.00391(14)
O1	96*h*	0.103808(4)	0.19787(4)	0.28057(4)	1	0.01011(13)
Li1	24*d*	0.375	0	0.25	0.674(5)	0.0343(16)
Li2	96*h*	0.146(2)	0.177(2)	0.438(2)	0.3375(14)	0.0196(11)
CZ-LLZO, *T* = 300 K, $Ia\bar{3}d$, Z = 8, *a* = 12.8775(2) Å,						
La1	24*c*	0.125	0	0.25	1.001(4)	0.00795(15)
Zr1	16*a*	0	0	0	0.456(3)	0.00529(12)
Ta1	16*a*	0	0	0	0.544(3)	0.00529(12)
O1	96*h*	0.10314(4)	0.19792(4)	0.28048(4)	1	0.01204(12)
Li1	24*d*	0.375	0	0.25	0.659(5)	0.0390(16)
Li2	96*h*	0.1511(4)	0.1737(4)	0.4372(4)	0.3413(13)	0.0231(12)
CZ-LLZO, *T* = 400 K, $Ia\bar{3}d$, Z = 8, *a* = 12.9051(2) Å,						
La1	24*c*	0.125	0	0.25	0.994(7)	0.0098(3)
Zr1	16*a*	0	0	0	0.454(3)	0.0075(2)
Ta1	16*a*	0	0	0	0.546(3)	0.0075(2)
O1	96*h*	0.10333(6)	0.19801(7)	0.28031(7)	1	0.0148(2)
Li1	24*d*	0.375	0	0.25	0.644(6)	0.051(4)
Li2	96*h*	0.1495(8)	0.1754(11)	0.4363(11)	0.3451(14)	0.032(3)
